# Experimental investigation and ANN modelling on CO_2_ hydrate kinetics in multiphase pipeline systems

**DOI:** 10.1038/s41598-022-17871-z

**Published:** 2022-08-11

**Authors:** Nagoor Basha Shaik, Jai Krishna Sahith Sayani, Watit Benjapolakul, Widhyakorn Asdornwised, Surachai Chaitusaney

**Affiliations:** 1 Artificial Intelligence, Machine Learning, and Smart Grid Technology Research Unit, Department of Electrical Engineering, Faculty of Engineering, Chulalongkorn University, Bangkok, 10330 Thailand; 2 School of Chemical and Bioprocess Engineering, University College Dublin, Belfield, Dublin, D04 V1W8 Ireland

**Keywords:** Chemical engineering, Energy

## Abstract

Gas hydrates are progressively becoming a key concern when determining the economics of a reservoir due to flow interruptions, as offshore reserves are produced in ever deeper and colder waters. The creation of a hydrate plug poses equipment and safety risks. No current existing models have the feature of accurately predicting the kinetics of gas hydrates when a multiphase system is encountered. In this work, Artificial Neural Networks (ANN) are developed to study and predict the effect of the multiphase system on the kinetics of gas hydrates formation. Primarily, a pure system and multiphase system containing crude oil are used to conduct experiments. The details of the rate of formation for both systems are found. Then, these results are used to develop an A.I. model that can be helpful in predicting the rate of hydrate formation in both pure and multiphase systems. To forecast the kinetics of gas hydrate formation, two ANN models with single layer perceptron are presented for the two combinations of gas hydrates. The results indicated that the prediction models developed are satisfactory as R^2^ values are close to 1 and M.S.E. values are close to 0. This study serves as a framework to examine hydrate formation in multiphase systems.

## Introduction

During the production of hydrocarbons, it is very common to encounter various contaminants and particles being transported in the flow line. When such flows are encountered, usually they fall under multiphase systems as there are many phases that concurrently exist in a flow^1^. Multiphase flow is come across in various domains. In the oil & gas and chemical processing industry, the multiphase flow has been of utmost importance, especially about the challenges that come across due to its occurrence of it in the flow lines^2–4^. The frequent transportation of natural gas is via subterranean pipelines, and immense attention has to be given to the safety of the gas transportation system. If the pipelines are exposed to extreme operational conditions like low temperatures when being operated at high pressures, we have many flow assurance issues^5,6^. One such issue is the occurrence of gas hydrates in the pipelines. Gas hydrates occur in production pipes in the subsea region and produce blockages in the pipelines eventually compromising the safety of the pipelines^7^.

**Figure 1:**
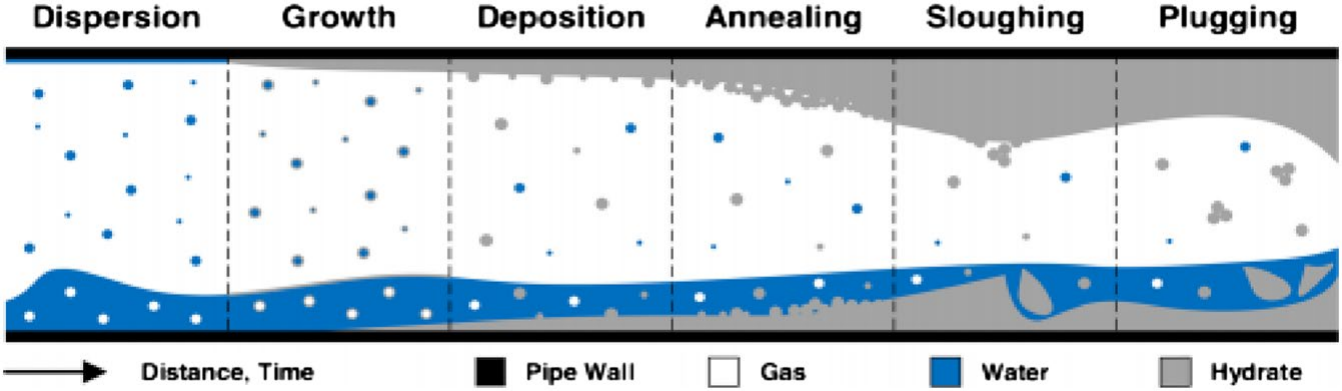
Growth of the hydrates in gas-dominant multiphase systems^8^.

Figure 1 depicts the growth notion of gas hydrate in gas dominant multiphase system.

Gas hydrates are solid ice-like crystalline compounds that are made up of polyhedral water voids^9,10^. When water and gases come into contact at a given temperature and pressure, hydrates are created. Water crystallizes with natural gases and related liquids in a ratio of 85 percent mole water to 15% hydrocarbons to generate gas hydrates. They can be found in both gas and gas/condensate wells as well as oil wells. The hydrates are three-dimensional frameworks that are formed with the participation of hydrogen bonding between the water molecules. Every cavity of the hydrate structure is either filled with a guest gas or could be void. The removal or prevention of the hydrate formation in the flowlines is very important; as mentioned earlier, they affect the safety of the operation alongside huge environmental and economical losses^11^. They play a major role in gas separation, fuel transportation, fuel storage and energy storage from a technological standpoint^12–14^. They could potentially be used as a source of fuel^15,16^.

**Figure 2:**
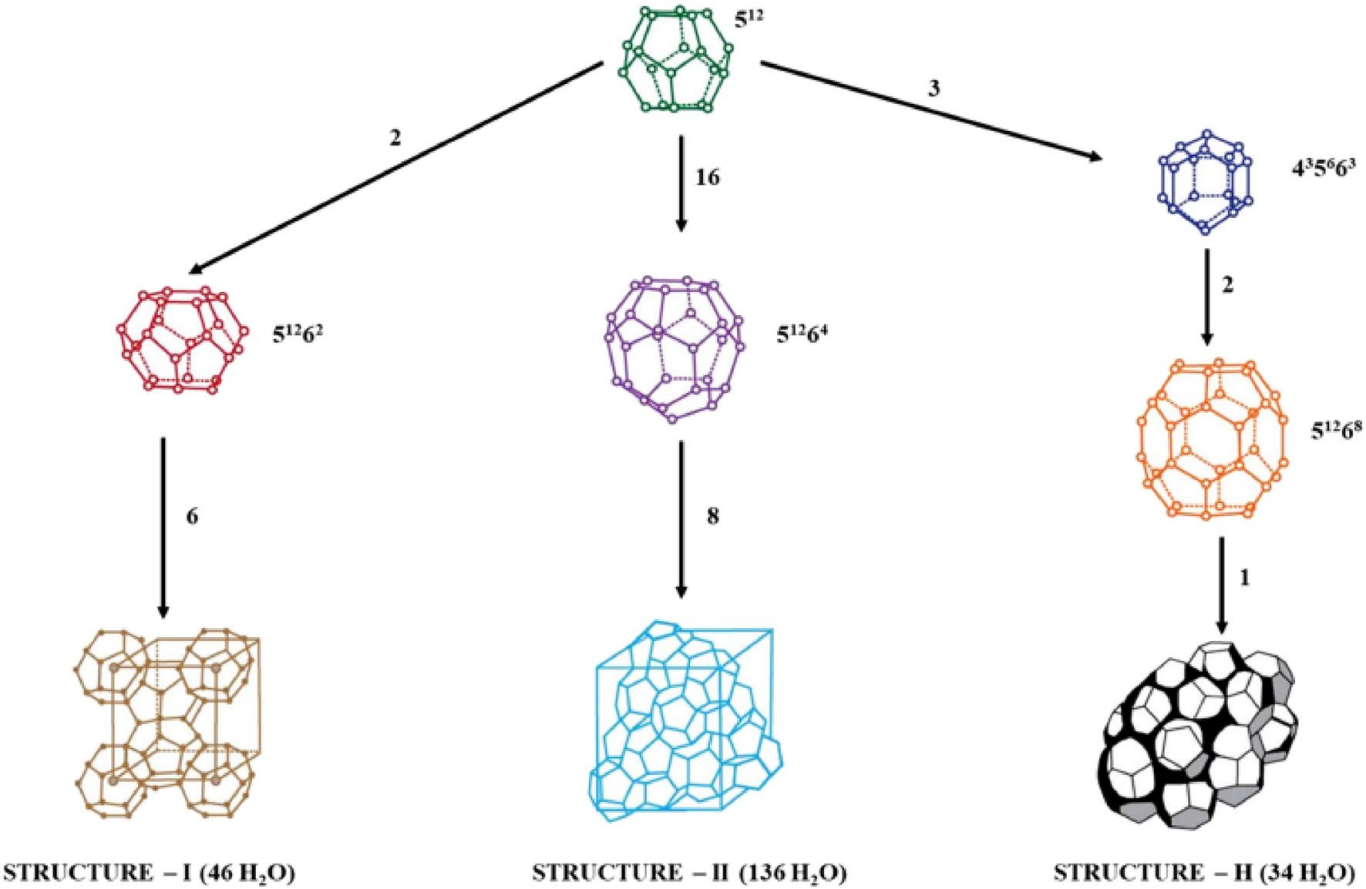
Standard structures of gas hydrates (sI, sII, sH)^17^.

Distinct types of guest molecules, as well as their sizes, result in different gas hydrate structure forms. Gas hydrates are distinguished by the shapes of their cavities and the distribution of those cavities inside a given cell. As shown in Fig. 2, there are three common hydrate structures that have been discovered to date.

As of now, gas hydrates have caused more issues than they have solved. The determination of gas hydrates by experiments and real-time situations is always not feasible due to high costs and danger prone to hydrate generation in closed systems. So, to counter this issue, several researchers over the years have developed and proposed many thermodynamic models and kinetic models that can predict the production of gas hydrates. Based on these results, many inhibitors are proposed, and the effectiveness of various gas hydrate inhibitors is studied^17,18^. The thermodynamics and kinetics of gas hydrate production should be investigated. Using considerable experimental and statistical data already accessible in the literature, the thermodynamic conditions of hydrate formation can now be predicted. However, there isn't enough research on hydrate formation kinetics, and the majority of experimental data and kinetic models in the literature are lacking.

Gas hydrate kinetics are known to be controlled by the nucleation time (induction time) and development of gas hydrate crystals. The nucleation time is the amount of time it takes for hydrate nuclei to reach a critical size and remain stable. Hydrate nuclei will develop and produce hydrate crystals once they have reached the threshold size. As a result, both nucleation and growth stages should be included in the hydrate kinetic study. However, it is assumed that the nucleation stage is a stochastic occurrence that is influenced by a number of factors, including the history of the water sample, and that it cannot be anticipated with certainty^19^. As a result, most hydrate formation kinetic models and research focus on the growth rate step because predicting the stochastic and ambiguous nucleation process is difficult and unpredictable.

Gas hydrate kinetics was first studied in the 1960s. Glew and Haggett^20^ created a kinetic model to study the dynamics of gas hydrate generation. Experiments demonstrated that hydrate production is an exothermic process and that the rate of growth is proportional to the temperature differential between the reactor and its cooling bath. As a result, the rate of heat transfer from the reactor to the cooling bath was regarded a regulating factor. The rate of hydrate formation was later estimated using experimental data and a simplified energy balance for the reactor.

Vysniauskas and Bishnoi's experimental results^21^ on the genesis of methane and ethane hydrates were explained by Englezos et al.^22^ using a kinetic model. The interfacial mass transfer was modeled using the two-film theory, while the kinetic model was based on crystallization theory. The driving mechanism for hydrate formation was identified as the fugacity differential between dissolved gas and three-phase equilibrium fugacity. Herri et al.^23^ used the light scattering technique to quantify the size distribution of crystals in situ in a stirred reactor of volume 1000 cm^3^ at constant pressure. The stirring speed (*ꞷ*), which should be taken into consideration in modeling, has been proven to have a significant impact on the kinetics of methane hydrate production. When a hydrate film forms at a water/gas contact, it expands laterally as well as in the direction normal to the interface, changing the thickness of the film. The lateral growth rate of hydrate can be calculated using the initial film thickness. By floating a single methane bubble in water, Li et al. used a microscope to calculate the initial thickness of a methane hydrate layer^24^. In addition, the morphological alteration of the hydrate coating covering the surface of the bubble was investigated.

Peng et al.^25^ studied the rate of hydrate film development as a function of the interface on the surface of a gas bubble suspended in water in the lateral and normal directions. In pure water, the lateral film development rate of pure and mixed gaseous hydrates was determined at various temperatures. They discovered that given the same driving force, the lateral growth rates of mixed-gas hydrate films were slower than pure gas hydrate films. If the governing equations must be determined, kinetic modeling of gas hydrate production is extremely difficult. The mechanics of hydrate formation is a bit hazy, and several driving forces may be at work during the process. Furthermore, the rate of growth is affected by composition and testing conditions. In this state, AI-based approaches can aid in the prediction of hydrate compound growth rate if some experimental data is provided. Mohammadi et al.^26–28^ provided a statistical model based on a feed-forward artificial neural network (ANN) algorithm that could predict the thermodynamic conditions of the hydrate systems: H_2_O + H_2_, H_2_O + H_2_ + THF, and H_2_ + H_2_O + tetra-n-butyl ammonium bromide. They showed that the projected and experimental results are in good agreement, proving the algorithm's reliability as a predictive tool.

Zahedi et al.^29^ used the Engineering Equation Solver (E.E.S.) and Statistical Package for the Social Sciences (SPSS) software to approximate hydrate formation temperature (H.F.T.) from the 203 experimental data points obtained from the literature. H.F.T. was also calculated using an ANN approach that used 70% of the experimental data for training. The spectacular estimate performance of ANN was proven when the results of the ANN model were compared to 30% of the testing data. When compared to traditional approaches, it was discovered that ANN was more accurate.

It can be observed clearly that the current modelling techniques are not accurate enough or don't have the feature of predicting the kinetics of gas hydrates, especially when a multiphase system is encountered. Importance of studying the hydrates is also critical, as mentioned earlier. In order to investigate the kinetics of gas hydrate formation in gas dominating multiphase pipelines, an experimental examination was carried out in this study. Following that, using ANN, two prediction models are proposed based on the outcomes.

## Artificial neural networks

ANNs were developed with the purpose of imitating the actions and functions of the nervous system and human brain with learning and memorizing capabilities to do a precise task. The ANN network receives independent variable inputs and manipulates them using internal mathematical operations to produce ANN network dependent variable outputs^30^. ANNs are composed of several interconnected processing components that "learn" to represent and extrapolate the correlations between the dependent and independent variables. When new data is received, ANNs use learning algorithms that may make changes or learn independently^31^. As a result, they are extremely valuable for non-linear statistical data modeling. An ANN typically has three or more connected layers. The first layer consists of input neurons; these neurons transmit data to the second hidden layer, which transmits the data to the final output layer. The hidden layers units seek to learn about the collected information by measuring it in compliance with the internal structure of the ANN^32^. These rules allow changing their output, which is subsequently passed to the next layer. Backpropagation is a technique that enables the ANN to alter its output results to compensate for errors. The output is identified as an error during the supervised training phase, and the information is relayed backward. Each weight is weighted according to how much it contributed to the inaccuracy. The error is used to modify the weight of the ANN's unit connections to account for the discrepancy between the predicted and actual outcomes^33^. The ANN will learn how to lower the chance of errors and undesired outcomes over time. ANNs learn by analyzing data sets, which aids in identifying the most cost-effective and optimal solutions while creating computation functions^34^.

## Methodology

### Experimental setup

**Figure 3:**
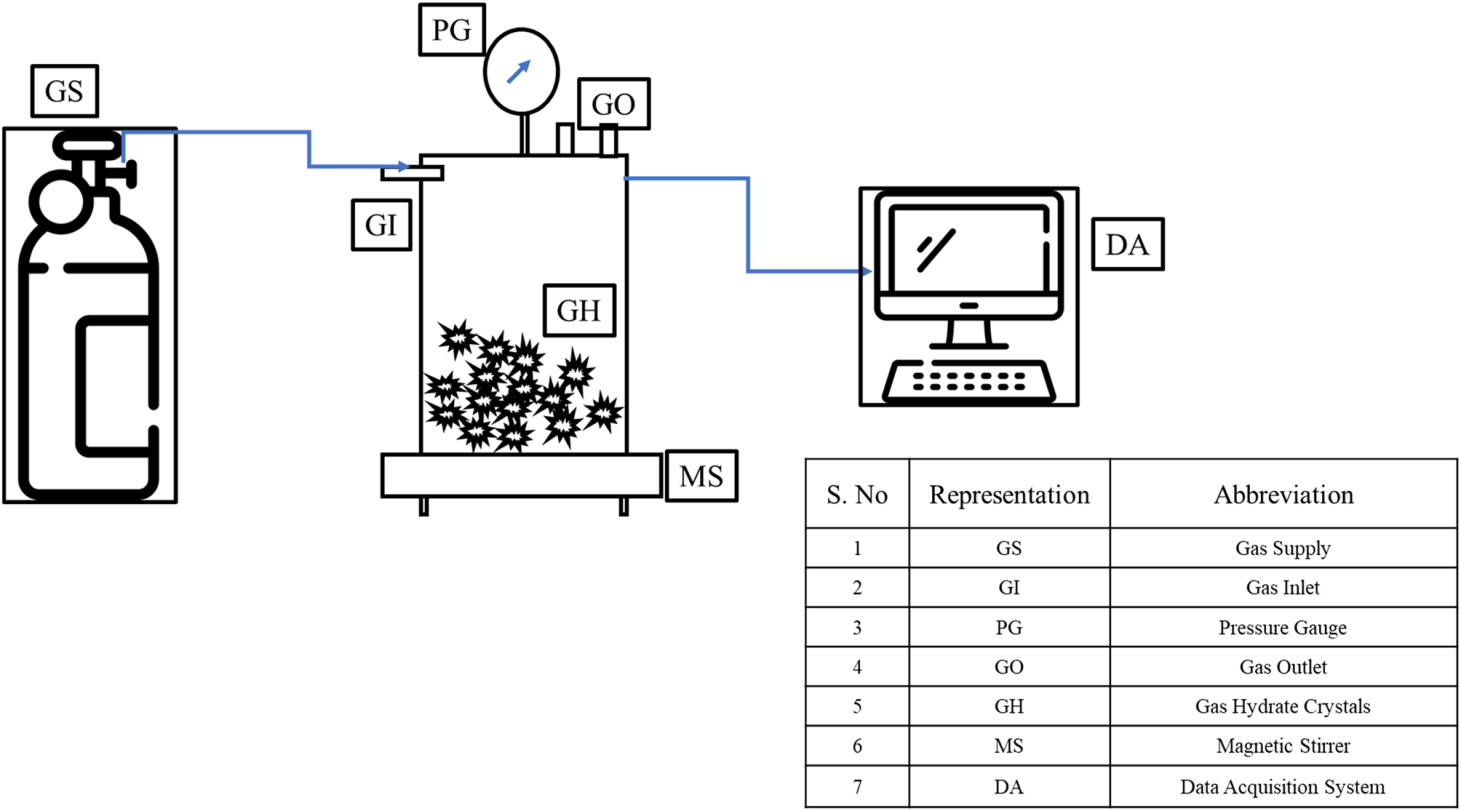
Experimental setup visualisation.

The experimental setup used in this study is shown in Fig. 3. The autoclave reactor used in this work is of volume 700 ml. It has been designed to be operated to the maximum pressure of 20 Mpa and in the temperature range of − 10 to 30 °C. The reactor is made out of stainless steel. The experimental setup is calibrated before starting the experiments, and it is found that it has reported the error of ± 0.3 °C and ± 0.7 bar. This uncertainty is less than 1% of the experimental range considered. For the experiments on the pure system, 85–15 Vol% is taken for the CO_2_ gas and deionized water, respectively. Similarly, 70–15–15 Vol% is used for the CO_2_ gas, crude oil, and deionized water for the multiphase system. The crude oil used in this work is pyrene crude oil. The details of the crude oil composition can be found in the literature^15^.

### Experimental analysis

Experiments are conducted in the pressure range of 2.5–3.5 MPa. A fresh sample (firstly, deionized water + CO_2_ gas system and later crude oil + CO_2_ gas + deionized water system) is placed in the reactor cell to determine the kinetics of gas hydrate production. The reactor cell is then dropped to 285 K, which is substantially above the temperature at which hydrates occur. When the agitating mechanism is turned on, the stirrer is activated, and the gas is permitted to dissolve into the multiphase mixture as needed. During this period, the system establishes equilibrium. The system is then cooled to 273.15 K with the agitation shut off. All kinetic tests were carried out at least three times, and the findings provided are averages.

**Figure 4:**
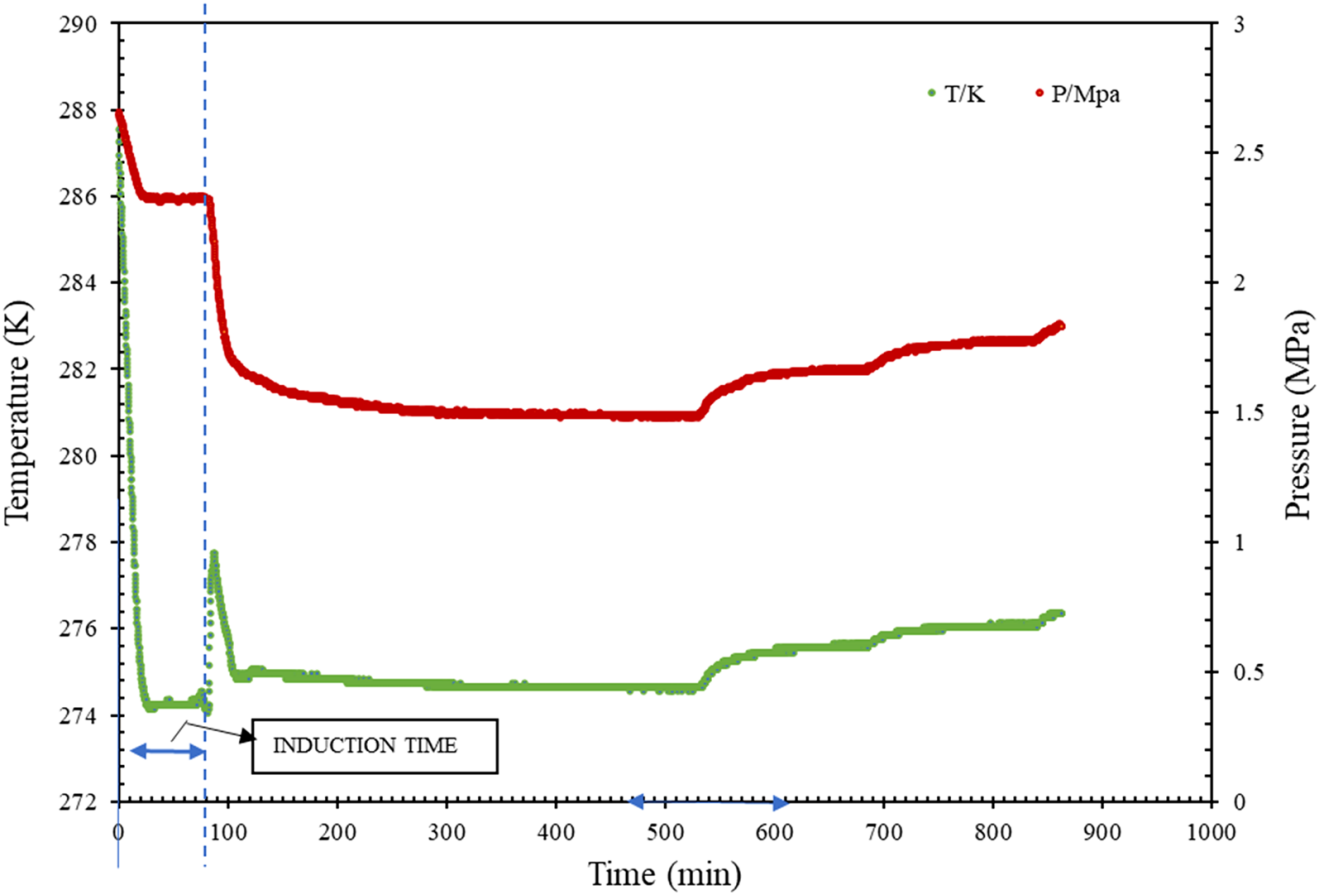
Pressure/temperature vs. time (*t*) curve to find induction time.

The induction time for the formation of the gas hydrates was chosen as the time required to start producing large numbers of hydrates. Because gas hydrate synthesis is an exothermic process for lowering pressure, the induction period is triggered by a rapid shift in temperature. It can be identified as the moment when a sudden pressure decrease occurs in conjunction with an increase in temperature, as seen in Fig. 4. The later parts of the curve are related to the dissociation of the hydrate due to increased pressure and temperature.

**Figure 5:**
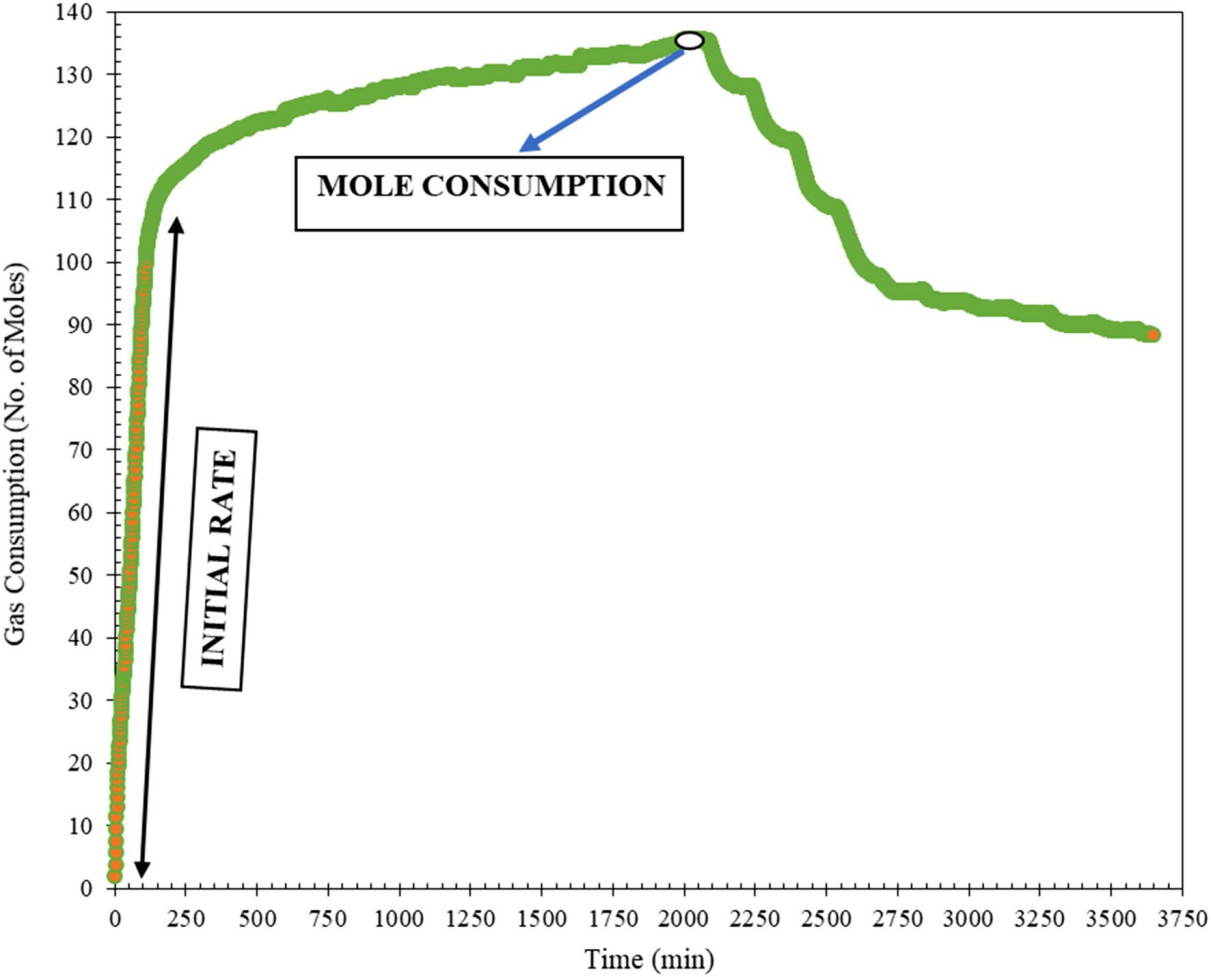
Gas consumption (CO_2_) curve vs. time (*t*).

Figure 5 represents the gas consumption (CO_2_) curve vs. time (*t*). The amount of carbon dioxide (CO_2_) used to conduct the experiment for maximum hydrate formation is a major stumbling block to commercializing gas hydrate technology. The following equation is used to calculate gas consumption. During the formation of gas hydrates, it is assumed that the volume of water does not vary. As a result, the following Eq. (1) is utilized for the isothermal experiment.

1 $$\Delta {n}_{g}= \frac{V}{R} \left[{\left(\frac{P}{ZT}\right)}_{0}-{\left(\frac{P}{ZT}\right)}_{t}\right]$$

where *P* and *T* are the multiphase system's pressure and temperature, respectively.

The universal gas constant is *R*, and the volume of the gas phase is V. The compressibility factor, Z, is determined by the PengRobinson equation of state. The subscripts 0 and *t* denote the start of the experiment and the experimental circumstances at time *t*, respectively.

As shown in Eq. (2) below, the gas uptake is determined to standardize the amount of gas consumed and eliminate the sample size. It denotes the amount of gas contained in one mole of water:

2 $${\varvec{U}}=\frac{\Delta {{\varvec{n}}}_{{\varvec{g}}}}{{{\varvec{n}}}_{{\varvec{w}}}}$$

where ${{\varvec{n}}}_{{\varvec{g}}}$ is the number of moles of gas and ${{\varvec{n}}}_{{\varvec{w}}}$ is the number of moles in water.

The promotion behavior of the hydrate formation can be easily identified with induction time^6^. The use of induction time alone can occasionally be deceptive because hydrate induction time measurements are stochastic in nature. So, in this work, the total number of moles consumed with resopect to time is also considered to analyse the kinetics of the chosen systems.

### ANN modelling

**Figure 6:**
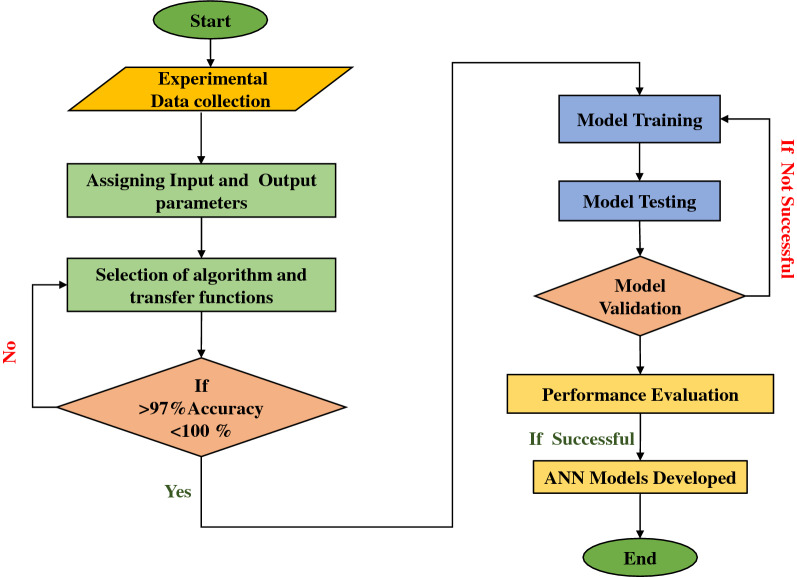
Overall proposed method for the developed ANN models.

The approach depicted in Fig. 6 follows the overall evolution of ANN models. ANN models are created using the experimental datasets. For the creation of ANN models, inputs such as time, pressure, and temperature are used, while outputs such as the number of moles and formation rate are used. Other parameters like volume of the fluid, stirring rate, volume of the reactor are not considered in this work. This is because, currently, the experiments conducted were with constant sample volume and non variable reactor. Also, for all the experiments, the stirring rate is maintained as constant. The data is split up into three categories: training, testing, and validation. The model is trained using the Levenberg Marquardt (LM) algorithm using a training dataset. The trained models are then tested with a testing dataset and validated with a validation dataset for better performance.

**Figure 7:**
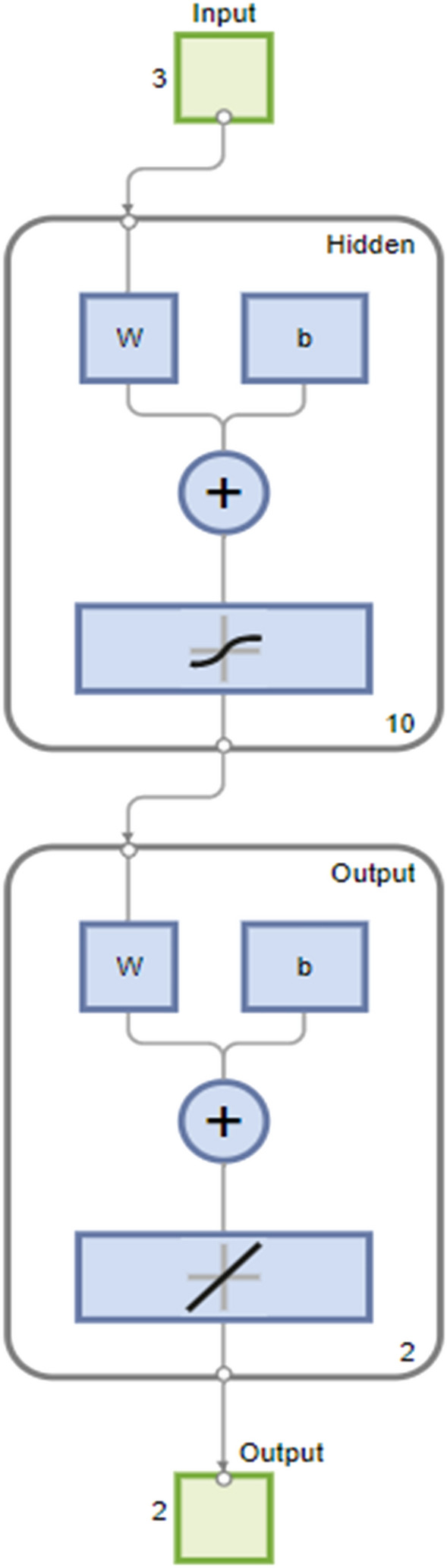
Proposed LM-based ANN architecture.

Using the 'trainlm' technique, many iterations were conducted during ANN model training. The weights involved in the 'trainlm' were successfully updated in a smoother and more general manner. The proposed network has three inputs (time, pressure and temperature), ten hidden neurons, and two outputs (no.of moles and formation rate). In other words, as shown in Fig. 7, the optimal ideal network design was determined to be 2-10-2. The LM algorithm is chosen in the present study as it solves non-linear least squares problems using a damped least-squares technique. These minimization problems are most noticeable in least-squares curve fitting. This method combines the Gauss–Newton algorithm and the gradient descent method. The LM approach is more durable than the GNA algorithm, which means it will frequently find a solution even if it begins very far from the final minimum.

## Results and discussion

### Experimental results

**Figure 8:**
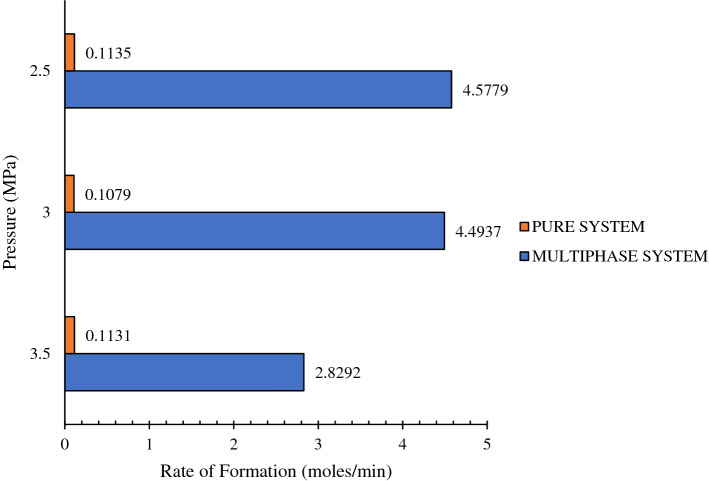
Kinetic performance of pure and multiphase systems.

The rate of hydrate formation is mentioned in Fig. 8. It can be noticed that the formation rate in multiphase systems is much higher when compared to that of the pure system. This means that the multiphase system containing crude oil and CO_2_ gas promotes gas hydrate formation. This is due to the presence of contaminants and lengthy carbon chains. The production of gas hydrates is influenced by the presence of longer carbon chains^15,35–37^. Also, at low temperature and high-pressure situations, crude oil behaves like a non-newtonian fluid. The CO_2_ gas is a non-polar gas that will be dissolved in crude oil which is a non-polar fluid^38–41^. This provides the opportunity for the gas to be more vividly available for the hydrates to form. The hydrate growth in the system starts as shown in Fig. 5. But the results discussed in Fig. 8 are based on the gas consumption in the whole process with respect to time.

### ANN modelling

**Figure 9:**
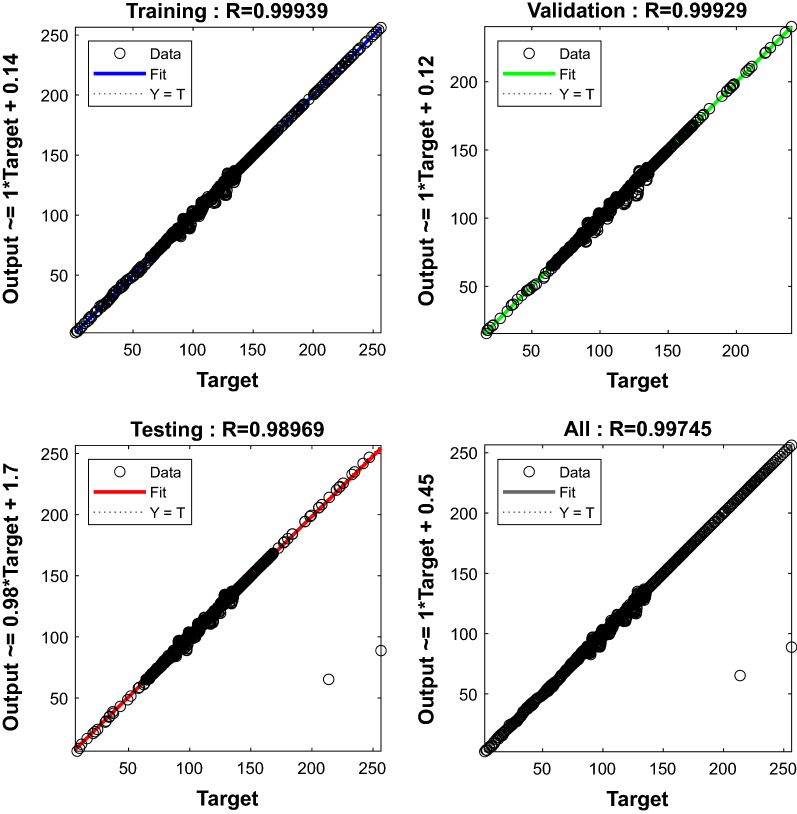
Regression plots during ANN training for gas + CO_2_.

**Figure 10:**
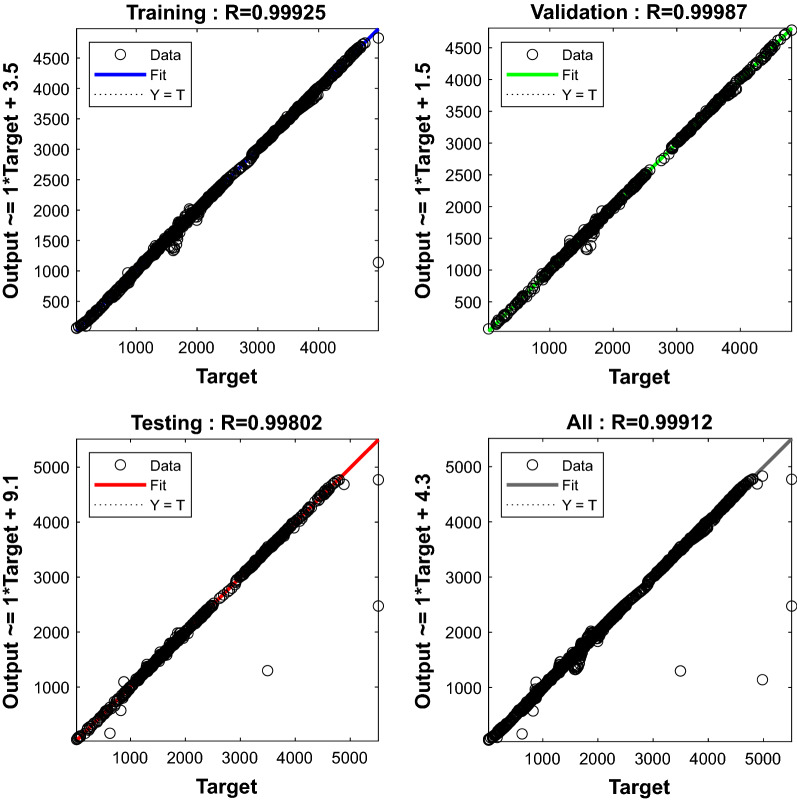
Regression plots during ANN training for gas + CO_2_ + crude oil.

The ANN learning challenge may be viewed as a function optimization process in which we strive to discover the appropriate network parameters to reduce network error. However, some function optimization approaches, such as the LM algorithm, may be directly applied to network learning. The LM algorithm provides a solution for minimizing a (usually nonlinear) function across a set of parameters for the function. The LM learning technique aims to describe a set of connections that produces a mapping that is well suited to the training set. Furthermore, because ANNs are highly nonlinear functions, the training issue may be treated as a generic function optimization problem, with the changeable parameters being the network's weights and biases, and the Levenberg–Marquardt principle can be directly used in this instance. The two ANN models are trained using the LM technique and the training datasets. R^2^ values for both ANN models are found to be close to 1.0, as illustrated in Figs. 9 and 10. The proposed ANN models predicted the training, validation, and testing data sets well, as shown in Figs. 9 and 10, with R^2^ of 0.9999, 0.99929, and 0.98969 for gas + CO_2_, and R^2^ of 0.99925, 0.9987, and 0.99802 for gas + CO_2_ + crude oil, respectively. It can be seen from testing datasets that are subsequently given to the trained models to ensure that they are performing as expected. The evaluated models yielded encouraging results, allowing the validation process to proceed. Validation datasets unknown to the models are used to validate the tested models.

Table 1 shows the M.S.E. and R^2^ values for all three stages of the ANN model creation process. Equations (3) and (4) are used to calculate the M.S.E. and R^2^ values. All M.S.E. and R^2^ values were determined to produce satisfactory results in all stages, with M.S.E. values near to 0 and R^2^ values close to 1.0.

**Table 1:** M.S.E. and R^2^ values recorded during ANN training.

Combination	Stage	M.S.E	R^2^
Gas + water system	Training	0.00374	0.99939
	Validation	0.0158	0.99929
	Testing	0.0161	0.98969
Gas + crude oil + water system	Training	0.0827	0.99925
	Validation	0.0155	0.99987
	Testing	0.018	0.99802

3 $$MSE = \frac{1 }{n} \sum_{i=1}^{n}{({y}_{i - }{\tilde{y}}_{i})}^{2}$$

4 $${R}^{2 }= 1- \frac{{SS}_{RES}}{{SS}_{TOT}} = 1- \frac{{\sum }_{i}{({y}_{i} - \hat{y})}^{2}}{{\sum }_{i}{({y}_{i} - {\bar{y}}_{i})}^{2}}$$

**Figure 11:**
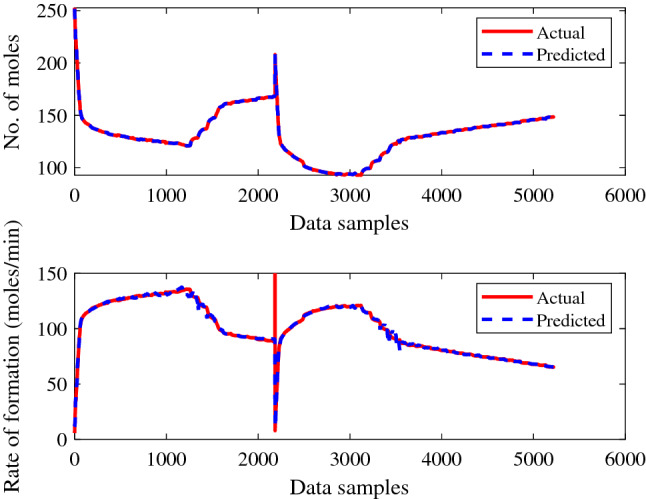
ANN training performance for gas + CO_2_.

**Figure 12:**
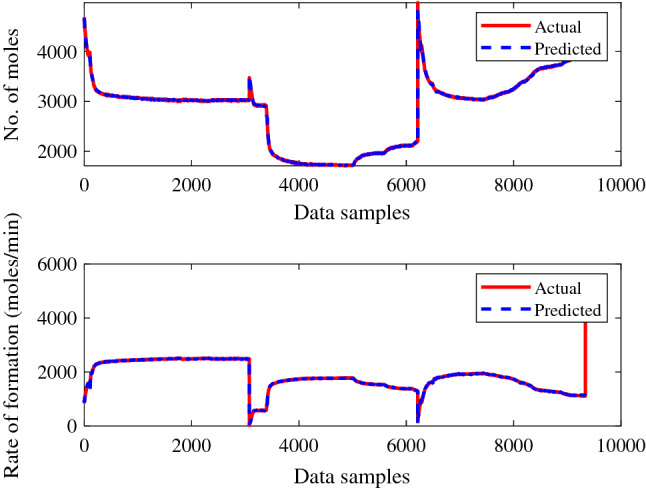
ANN training performance for gas + CO_2_ + crude oil.

**Figure 13:**
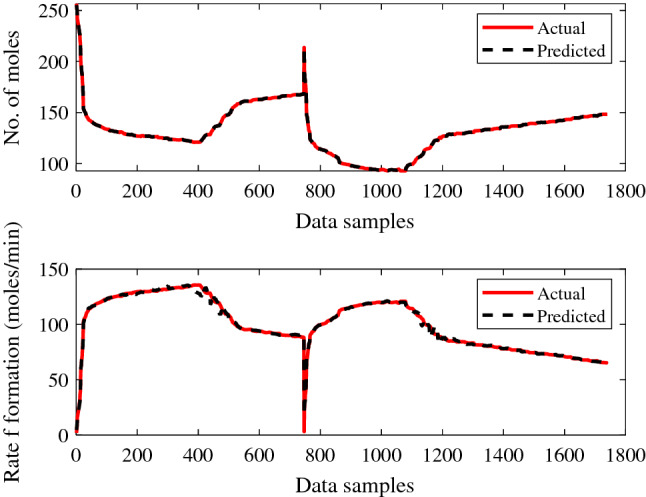
ANN testing performance for gas + CO_2_.

**Figure 14:**
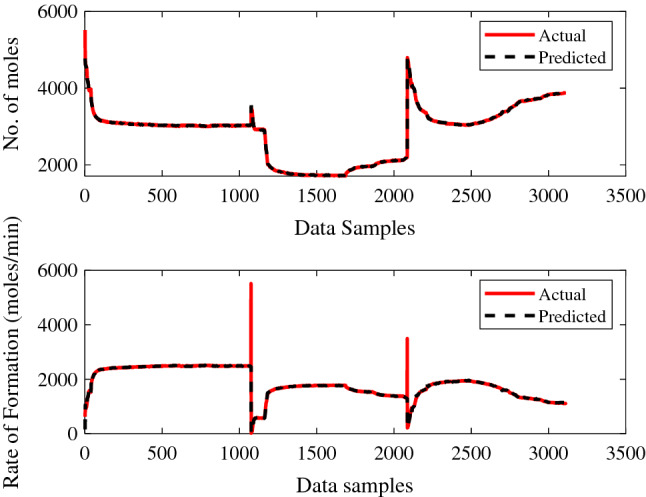
ANN testing performance for gas + CO_2_ + crude oil.

Figures 11 and 12 show the training performance of the created ANN models for gas + CO_2_ and gas + CO_2_ + crude oil, which were in excellent agreement for predicting the supplied experimental data sets. It is clear from the training results of the created ANN models, as shown in Figs. 11 and 12, how successfully the prediction is made. Similarly, the testing performance of the model was shown to be adequate for the set of experimental values supplied, as represented in Figs. 13 and 14.

The trained ANN models performed better during the testing phase, when making predictions against given experimental values. The suggested method then had the best performance for the provided data samples and made the fewest errors. Figures 13 and 14, shows the higher performance of a trained ANN models during the prediction of each experimental value may be noticed. The recommended strategy produced the least error with improving performance for the given data.

**Figure 15:**
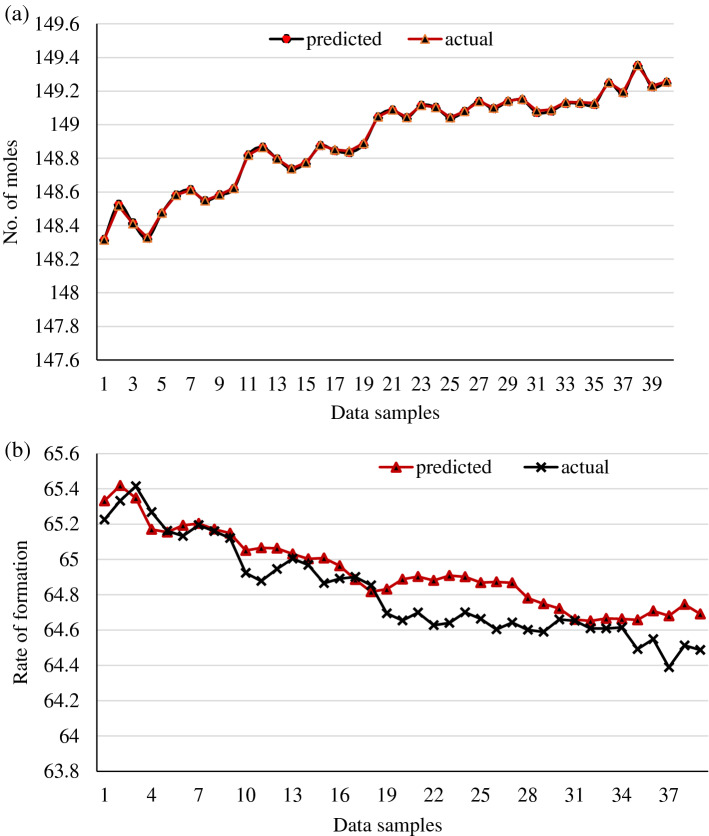
Validation plot for gas + CO_2_.

**Figure 16:**
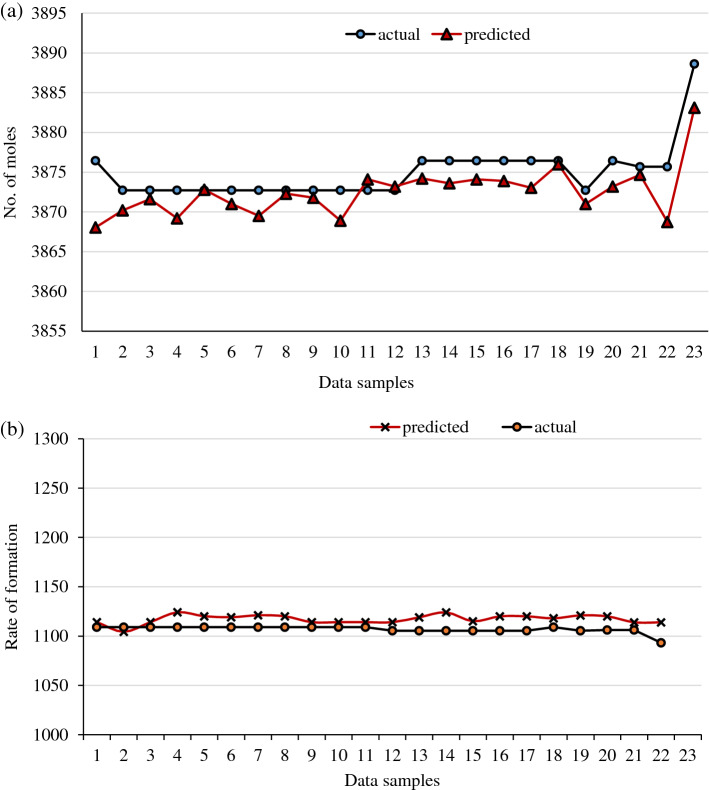
Validation plot for gas + CO_2_ + crude oil.

During the validation stage, the tested models are given a random set of parameters to forecast the number of moles and rate of deformation. The models projected correctly, with 98.37 and 97.86% prediction accuracy, respectively. Figures 15 and 16 exhibit graphic representations of the validation plots for the number of moles and deformation rate.

The same process is followed with crude oil data during the validation. The validation plots are represented graphically, as shown in Figs. 15 and 16. The plots indicated promising results with prediction accuracies of 98.3 and 99.10 percent, respectively. To sum up, the best performances found at the validation stage, associated with a high numerical prediction performance. It showed that the proposed ANN models forecasted experimental data sets very well and this framework can be utilized in the predicting gas hydrate formation in multiphase systems.

## Conclusions

In this work, Artificial Neural Networks (ANN) are developed to study and predict the effect of the Multiphase system on the kinetics of gas hydrates formation. Primarily, a pure system and multiphase system containing crude oil are used to conduct experiments. The details of the rate of formation for both systems are found. Then, these results are used to develop an A.I. model that can be helpful in predicting the rate of hydrate formation in both pure and multiphase systems. To forecast the kinetics of gas hydrate formation, two ANN models with Single Layer Perceptron (S.L.P.) are presented for the two combinations of gas hydrates.

From the experimental analysis, it was observed that the addition of the crude oil system influences the mole consumption and rate of formation, which are key in terms of the kinetics of the gas hydrates. It can be clearly observed that the multiphase system tends to form the hydrate much faster compared to the simple system. This is due to the non-Newtonian behaviour of crude oil at high pressure and low-temperature conditions. During these situations, crude oil allows more gas dissolution, resulting in higher gas availability for hydrate formation. Further, the results indicated that the prediction models developed are satisfactory as R^2^ values are close to 1 and M.S.E. values are close to 0. This work helps as a platform for the efficient application of ANN modelling to determine the hydrate formation in multiphase systems as the evaluation of hydrates in multiphase systems is very complex. If the datasets are smaller, the suggested approach might not be trustworthy. Therefore, it is advised to take into account as much as large as possible to forecast the formation of gas hydrates. The work would be further extended in future by involving various other gases and gas mixtures.

## Data Availability

The datasets used and/or analysed during the current study are available from the corresponding author on reasonable request.
